# Proprioceptive Feedback Facilitates Motor Imagery-Related Operant Learning of Sensorimotor β-Band Modulation

**DOI:** 10.3389/fnins.2017.00060

**Published:** 2017-02-09

**Authors:** Sam Darvishi, Alireza Gharabaghi, Chadwick B. Boulay, Michael C. Ridding, Derek Abbott, Mathias Baumert

**Affiliations:** ^1^School of Electrical and Electronic Engineering, University of AdelaideAdelaide, SA, Australia; ^2^Division of Functional and Restorative Neurosurgery, and Centre for Integrative Neuroscience, Eberhard Karls University TuebingenTubingen, Germany; ^3^The Ottawa Hospital Research Institute, University of OttawaOttawa, ON, Canada; ^4^The Robinson Research Institute, University of AdelaideAdelaide, SA, Australia

**Keywords:** operant conditioning, reinforcement learning, brain-robot interface, brain-machine interface, brain-computer interface, beta rhythms, neurorehabilitation, stroke

## Abstract

Motor imagery (MI) activates the sensorimotor system independent of actual movements and might be facilitated by neurofeedback. Knowledge on the interaction between feedback modality and the involved frequency bands during MI-related brain self-regulation is still scarce. Previous studies compared the cortical activity during the MI task with concurrent feedback (MI *with* feedback condition) to cortical activity during the relaxation task where no feedback was provided (relaxation *without* feedback condition). The observed differences might, therefore, be related to either the task or the feedback. A proper comparison would necessitate studying a relaxation condition with feedback and a MI task condition without feedback as well. Right-handed healthy subjects performed two tasks, i.e., MI and relaxation, in alternating order. Each of the tasks (MI vs. relaxation) was studied with and without feedback. The respective event-driven oscillatory activity, i.e., sensorimotor desynchronization (during MI) or synchronization (during relaxation), was rewarded with contingent feedback. Importantly, feedback onset was delayed to study the task-related cortical activity in the absence of feedback provision during the delay period. The reward modality was alternated every 15 trials between proprioceptive and visual feedback. Proprioceptive input was superior to visual input to increase the range of task-related spectral perturbations in the α- and β-band, and was necessary to consistently achieve MI-related sensorimotor desynchronization (ERD) significantly below baseline. These effects occurred in task periods without feedback as well. The increased accuracy and duration of learned brain self-regulation achieved in the proprioceptive condition was specific to the β-band. MI-related operant learning of brain self-regulation is facilitated by proprioceptive feedback and mediated in the sensorimotor β-band.

## Introduction

When motor learning via physical practice (Doyon and Benali, 2005; Halsband and Lange, 2006; Malouin et al., 2013) is compromised due to motor deficits following stroke, motor imagery (MI) may provide an alternative training modality (Halsband and Lange, 2006; Boe et al., 2014). MI leads to improved cortical facilitation and reduced intracortical inhibition, even though the respective amplitudes achieved by MI may be reduced in comparison to motor execution (Clark et al., 2004; Léonard and Tremblay, 2007; Kumru et al., 2008). MI activates the sensorimotor system independent of actual movements (Gao et al., 2011; Szameitat et al., 2012) and may be facilitated by neurofeedback contingent to the event related changes in the oscillatory activities i.e., ERD/ERS (Bauer and Gharabaghi, 2015a; Pichiorri et al., 2015; Vukelic and Gharabaghi, 2015a). Such a feedback may be provided via visual or proprioceptive input (Bai et al., 2014; Boe et al., 2014; Vukelic et al., 2014; Bauer et al., 2015; Vukelic and Gharabaghi, 2015a).

Physiological knowledge of the impact of feedback modality on operant learning of the MI task, i.e., brain self-regulation is, however, still scarce. A detailed exploration would necessitate a refined study design for disentangling the contribution of the task condition and the feedback modality, separately: Previous studies compared the cortical activity during the MI task with concurrent feedback (MI *with* feedback condition) to cortical activity during the relaxation task where no feedback was provided (relaxation *without* feedback condition). The observed differences might, therefore, be related to either the task or the feedback. A proper comparison would necessitate studying a relaxation condition *with* feedback and a MI task condition *without* feedback as well.

Moreover, particularly proprioceptive input, e.g., by passive movement, is known to modulate the ongoing cortical activity in itself in a similar way to MI, but independently of any volitional brain modulation (Salenius et al., 1997; Pfurtscheller et al., 2002; Muller-Putz et al., 2007; Reynolds et al., 2015). However, previous studies (Gomez-Rodriguez et al., 2011) explored the effect of proprioceptive input on brain oscillations *during* the feedback period, which potentially clouds the MI-related cortical activity by the additional input of the feedback modality. Thus, knowledge on task-related cortical activity independent of the feedback period, i.e., during MI without feedback, is necessary.

We therefore compared visual and proprioceptive feedback during either sensorimotor MI-related desynchronization or relaxation-related synchronization. Furthermore, we applied a delayed feedback onset paradigm to study the cortical activity over the sensorimotor cortex in the absence/presence of feedback provision.

## Methods

### Ethics statement

The study conformed to principles outlined in the Declaration of Helsinki and was approved by the local human ethics committee of the University of Adelaide. All participants gave their written informed consent to participate in the study and all recorded data were de-identified.

### Participants

In this study, 10 able-bodied participants (four females, six males) aged 24–40 years were recruited. Participants were asked to remain alert, immobile, and to concentrate during the trials. The different types of MI, i.e., visual and kinesthetic MI, were explained to the participants and it was explicitly stated that the participants were expected to perform kinesthetic MI only. Participants were asked to minimize head and facial movements, swallowing, and blinking during the signal recording. They were given break periods to relaxation or move between consecutive runs when necessary.

### Brain-interface system

For data acquisition, a 72-channel Refa TMSi EXG amplifier, containing 64 unipolar and 8 bipolar channels and a 64-channel Waveguard EEG cap were used. The EEG data were recorded from 8 channels (F3, F4, T7, C3, Cz, C4, T8, and Pz) positioned according to the *international 10–20 system* of electrode placement. The AFz channel was used as the ground channel based on the recommendation of the manufacturer. The impedance between electrodes and the scalp was kept below 50 kΩ and this is sufficient due to the amplifier input impedance in the order of tera-Ohms (Volosyak et al., 2010). The amplifier does not require a reference channel as it uses built-in common average referencing of the recorded channels. It also disregards any electrodes with very high impedance (more than 256 kΩ) and excludes them from the common average reference. The signals were digitized at 1024 Hz and were then passed through a 50 Hz notch filter (3rd order Chebyshev) followed by a band pass filter (1st order Butterworth) with corner frequencies set to 0.1 and 49 Hz. Note that the built-in bandpass filter within the BCI2000 software is a first-order Butterworth filter that has a slower roll-off compared to a Chebyshev filter or an elliptic filter. Accordingly, the band pass filter with cut-off frequencies at 0.1 and 49 Hz did not filter out the 50 Hz power line noise completely. Therefore, a 50 Hz notch filter (3rd order Chebyshev) was applied as well to further suppress the power line noise.

Two orthoses (one for each hand) were designed to passively flex four fingers incrementally following the MI of the target hand. Each orthosis comprised a mechanical structure made of PVC and a Blue Bird BMS-630 servomotor. The control commands for servomotors were generated through the customized software and then translated to the servomotors of each orthosis using a Micro Maestro servo controller module.

The adopted software was a customized version of the BCI2000 Cursor Task (Schalk et al., 2004). The source code was modified to provide auditory commands. The application module of the software was also modified to update the position of the servomotors. The Micro Maestro servo controller received an updated command simultaneously with every cursor update on the monitor.

### Experimental design

To calculate event-related desynchronization (ERD) (Pfurtscheller and Lopes da Silva, 1999), the spectral power during MI performance is typically compared to the spectral power of a relaxation period before MI. However, since the relaxation period before MI occurs after a preceding MI trial, it may be affected by MI “after-effects,” such as slowly damping β-synchronization (Pfurtscheller, 2001; Pfurtscheller et al., 2005). Moreover, in classical setups for neurofeedback training, MI periods are paralleled by feedback while relaxation periods are usually not. The impact of the concurrent feedback may potentially cause cortical activities unrelated to MI. To overcome these limitations, we chose a specific paradigm in the present study characterized by the following features: (i) Disentangling MI and relaxation phases into two different tasks and providing both of them with feedback. (ii) Performing each of these tasks with a delayed feedback onset design to study the cortical activity in the absence/presence of feedback provision. (iii) Comparing different feedback modalities (proprioceptive vs. visual).

The delayed feedback onset design divides each task in a phase before feedback provision and a phase after feedback provision; these phases are referred to as *without* and *with* feedback, respectively, for the sake of simplicity, even though the former phase (“without”) is followed by the feedback phase (“with”). Importantly, the task phase before feedback onset (“without”) will allow capturing fast operant learning of brain self-regulation of intrinsic sensorimotor oscillations without concurrent extrinsic interferences.

The proprioceptive feedback modality was indeed a combination of visual and proprioceptive feedback as participants were instructed to observe the finger movement. Thus, visual feedback was provided in both paradigms, and thereby, any difference in performance between different setups is most likely related to provision of proprioceptive feedback. Therefore, for the sake of simplicity, the setup that provided combined proprioceptve and visual feedback is regarded as the “proprioceptive condition,” while the other setup that provided visual feedback only is referred to as the “visual condition.” Furthermore, a potential bias regarding action observation cannot be excluded when providing feedback during the relaxation task.

Every participant attended two sessions: one screening session and one online feedback session. During the screening sessions the optimum features of the EEG that resulted in the highest discrimination between MI and relaxation trials of each participant were defined. Then the defined optimum features of each participant's MI were used in the subsequent neurofeedback session. Eight out of 10 participants (four females, four males) who had EEG signals with distinctive features during hand MI passed the screening test (see below) and attended the neurofeedback training session.

#### Screening session

In the screening session, the subjects were asked to perform three runs of motor imagery, i.e., hand flexion of all four fingers, with 20 trials per run (**Figure 2**). Each trial started with an auditory command of “left” or “right” in parallel with a matching visual stimulus on a screen in front of the participant provided as an arrow pointing to the left or right. Each run included 10 trials of right and left motor imagery, respectively, in a randomized order without feedback provision; each trial lasted for 3 s and was followed by 3 s of relaxation. During relaxation trials participants had to stop MI and concentrate on their breathing. For further details on the time course of the screening session refer to Darvishi et al. (2015). The instruction to concentrate on breathing was given to ensure that (i) participants switch between MI and relaxation at the right time, and (ii) to provide participants with a tangible example of mental relaxation.

Previous studies (Pfurtscheller et al., 1997) demonstrated that performance of hand MI leads to a decrease in the spectral power of sensorimotor rhythms, i.e., event related desynchronization (ERD), followed by an increase in their spectral power, i.e., event related synchronization (ERS). In most participants these phenomena occur in the contralateral sensorimotor area within the α (8–13 Hz) and β (18–26 Hz) frequency bands. Thus, MI of the right or left hand is expected to lead to ERD followed by ERS in channels C3 or C4, respectively. However, some participants exhibited concurrent ERD over both contralateral and ipsilateral hemispheres during MI (Pfurtscheller et al., 1997).

Therefore, we searched for the occurrence of either unilateral or bilateral ERD during the (MI) trials of the screening session. The spectral power in each 2-Hz-wide frequency bin within α and β-frequency bands of the imagery and relaxation trials were compared to determine the combination of tasks that maximized the coefficient of determination (*r*^2^, representing the proportion of the single-trial variance in power that is due to the task). Right vs. left hand MI often causes the highest discrimination in sensorimotor rhythms. However, we only considered right vs. relaxation and left vs. relaxation in this study to minimize the cognitive load and fatigue. Thereby, we compared the *r*^2^-value between right/relax and left/relax combinations and selected the combination that maximized the *r*^2^-value. Thus, channel selection was made based on the pattern of ERD occurrence (contralateral or bilateral) and task selection, i.e., right vs. relax or left vs. relax, with the aim of a maximum *r*^2^-value. According to the mentioned guidelines, the optimum channel (s), i.e., C3 and/or C4, and the center of the optimum frequency bins selected for the neurofeedback sessions were operator independent.

After analyzing the screening session data, two participants revealed *r*^2^ < 0.05 and were, therefore, excluded from the study. Among those eight participants who demonstrated *r*^2^-values larger than 0.05, six participants (P1–P4, P7, P8) exhibited contralateral ERD, while two participants (P5, P6) revealed simultaneous ERD over both C3 and C4 channels (Table 1). For these two subjects with bilateral ERD, we provided the same weights to these features by averaging both ipsi- and contralateral ERDs.

**Table 1:** **Results of the screening session indicating the optimum side of imagined hand movement, channels and frequency bands for each participant, and number of runs performed in the following training session**.

**Participants**	**Side of imagined hand movement**	**Channels**	**Frequencies (Hz)**	**Number of runs**
P1	Left vs. Relax	C4	18	4
P2	Right vs. Relax	C3	13	4
P3	Right vs. Relax	C3	15	4
P4	Right vs. Relax	C3	17	8
P5	Right vs. Relax	C3, C4	15, 15	8
P6	Right vs. Relax	C3, C4	12, 18	4
P7	Left vs. Relax	C4	15	8
P8	Right vs. Relax	C3	15	8

#### Neurofeedback training session

**Figure 1:**
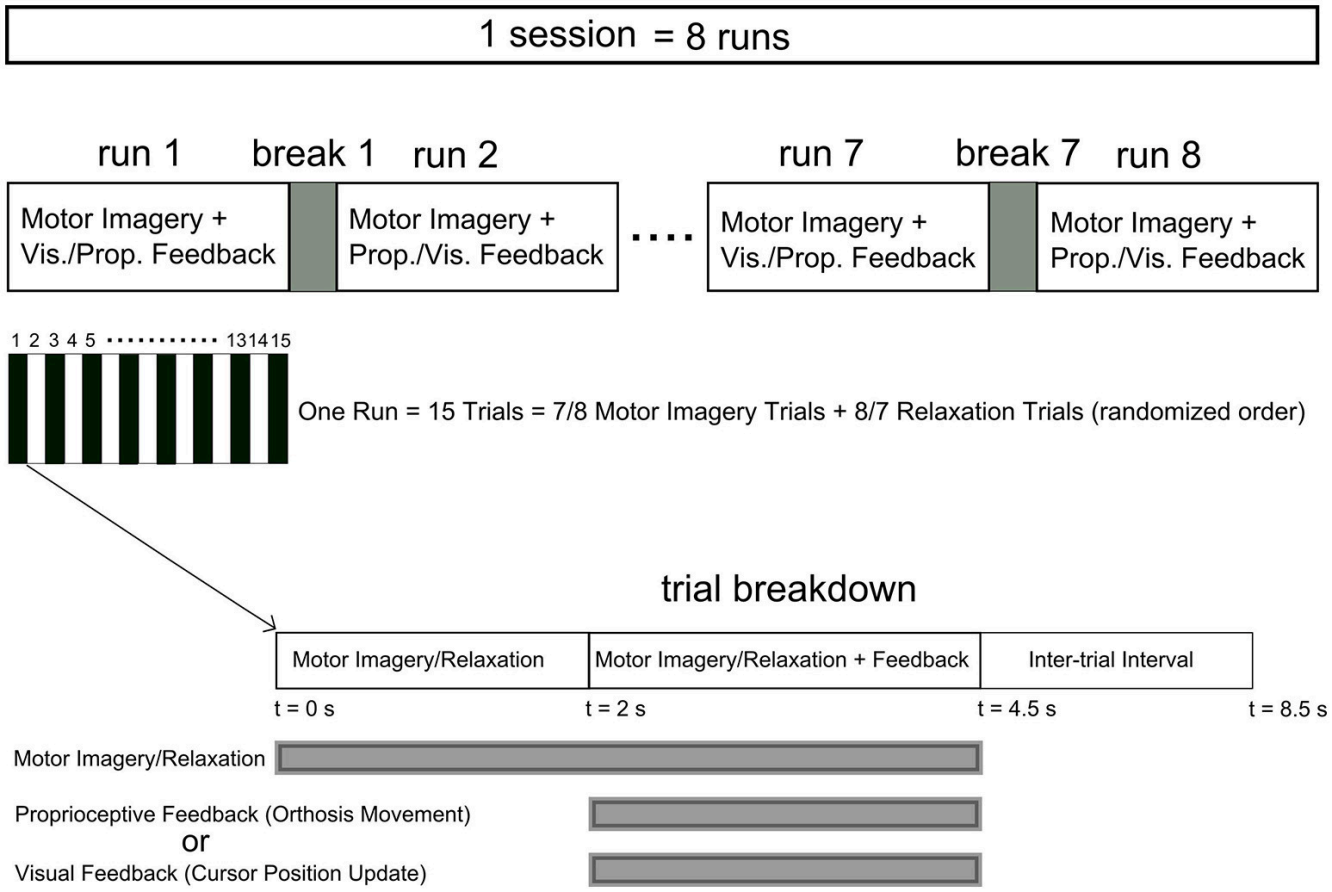
**Time course of the neurofeedback training sessions**. Each session comprised at least four runs (eight runs for P4, P5, P7, and P8) with a 2 min break between runs. Each run included 15 trials with relaxation and imagery trial performed in a randomized order. The feedback modality was interleaved between visual and proprioceptive across consecutive runs. Each trial started with a 2 s interval of imagery/relaxation without feedback followed by a 2.5 s section during which real time visual or proprioceptive feedback was provided. Following a 4 s inter-trial interval the next trial started.

The eight participants who passed the screening criteria were invited to return for an online feedback session within 2 weeks of their screening session. During the training session, four runs of MI of right/left hand four-finger flexion were performed. Those participants, who were willing to continue after four runs, participated in additional four runs (Table 1). Feedback modality (proprioceptive or visual) was interleaved over consecutive runs and there was a 2-min break between runs. Each run included 15 trials with 8 or 7 MI and 7 or 8 relaxation trials, respectively, which were sequenced randomly. Figure 1 illustrates the neurofeedback training time course.

In runs with visual feedback, at the start of each trial a red rectangle was displayed as a target at either the upper or lower half of the right side of the monitor, simultaneously with auditory commands indicating “relaxation” or “right” (“left” for P1 and P7), respectively. After 2 s, a cursor was shown on the middle of the left side of the monitor to indicate the start of the feedback period. The cursor was then moved horizontally from left to right with a constant speed within the next 2.5 s. Every 24 ms (105 times in 2.5 s), the vertical position of the cursor was updated depending on the classification result. The cursor went up and down during ERS and ERD, respectively. After reaching the right side of the screen, either with a hit or miss of the target, the next trial started after a 4 s inter-trial interval. For further details on cursor position updates we refer to Schalk and Mellinger (2010).

**Figure 2:**
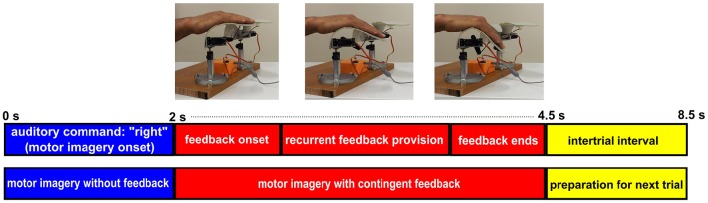
**Time course of a MI trial for right hand finger flexion with BRI**. Each imagery trial starts with a 2 s period of right hand finger flexion imagery without feedback provision. Then at *t* = 2 s, contingent proprioceptive feedback is provided through stepwise flexion of the orthosis. The feedback section lasts for 2.5 s and at *t* = 4.5 s the trial ends and after a 4 s interatrial interval the next trial starts.

In runs with proprioceptive feedback, however, participants sat in an armchair with their target hand placed on the orthosis (e.g., right hand on right orthosis) and their non-target hand placed on the armrest. For imagery trials (“right” or “left”), participants received proprioceptive feedback through congruent passive flexion of their target hand by the orthosis. For “relaxation” trials, participants received feedback by observing the flexion of the non-target orthosis with no hand engagement. This feedback design was intentionally chosen to overcome inherent limitations of providing *real* proprioceptive feedback for relaxation (see the discussion section for further details). In short, the setup with proprioceptive feedback studied here was intended for stroke rehabilitation and thus had to avoid feedback that might be counter-intuitiive. For simplicity, this condition will, however, be referred to as the proprioceptive condition as well in contrast to the visual condition where feedback for “relaxation” was provided by the position of a cursor only. The auditory command, i.e., “relaxation” or “right”/”left,” initiated the trial. After 2 s, the feedback period was commenced by instantly returning the orthosis to the fully extended start position. Every 24 ms, the orthosis flexed, if ERD was detected (see section Signal Processing), with up to 105 incremental flexions per 2.5 s feedback period. At the end of each feedback period, a “beep” signaled an inter-trial break and the next trial started after 4 s. Figure 2 illustrates the time course of a MI trial for right hand finger flexion with proprioceptive feedback.

### Signal processing

For online signal processing during the training session, a large Laplacian (LLP) spatial filter was used to derive surrogate channels C3-LLP and C4-LLP. The maximum entropy method (Marple, 1987) (MEM) was employed to define the autoregressive (AR) model of the EEG data. Using an 20th order AR model, the spectral power of the most recent 500 ms was estimated at the subject-specific frequencies and electrode positions determined from the screening session. To minimize the occurrence of false positives in the classifier, the following normalization procedure was adopted: (i) the spectral power of the most recent 18 s period of imagery and relaxation trials (equally represented) were buffered and continuously updated; (ii) the average and standard deviation of the buffer contents were calculated; (iii) every calculated spectral power component using the AR model was normalized by subtraction of the buffer's average followed by division of the buffer's standard deviation; (iv) if the normalized spectral power was negative, it was classified as ERD, whereas positive values were considered as relaxation. Normalized classifier outputs were used to update either the vertical velocity of the cursor on the monitor (visual feedback) or the flexion angle of the orthosis (proprioceptive feedback) every 24 ms. The chosen value for feedback update rate (i.e., every 24 ms) was adopted on the basis of a previous study of our group (Darvishi et al., 2013).

### Performance measures

**Figure 3:**
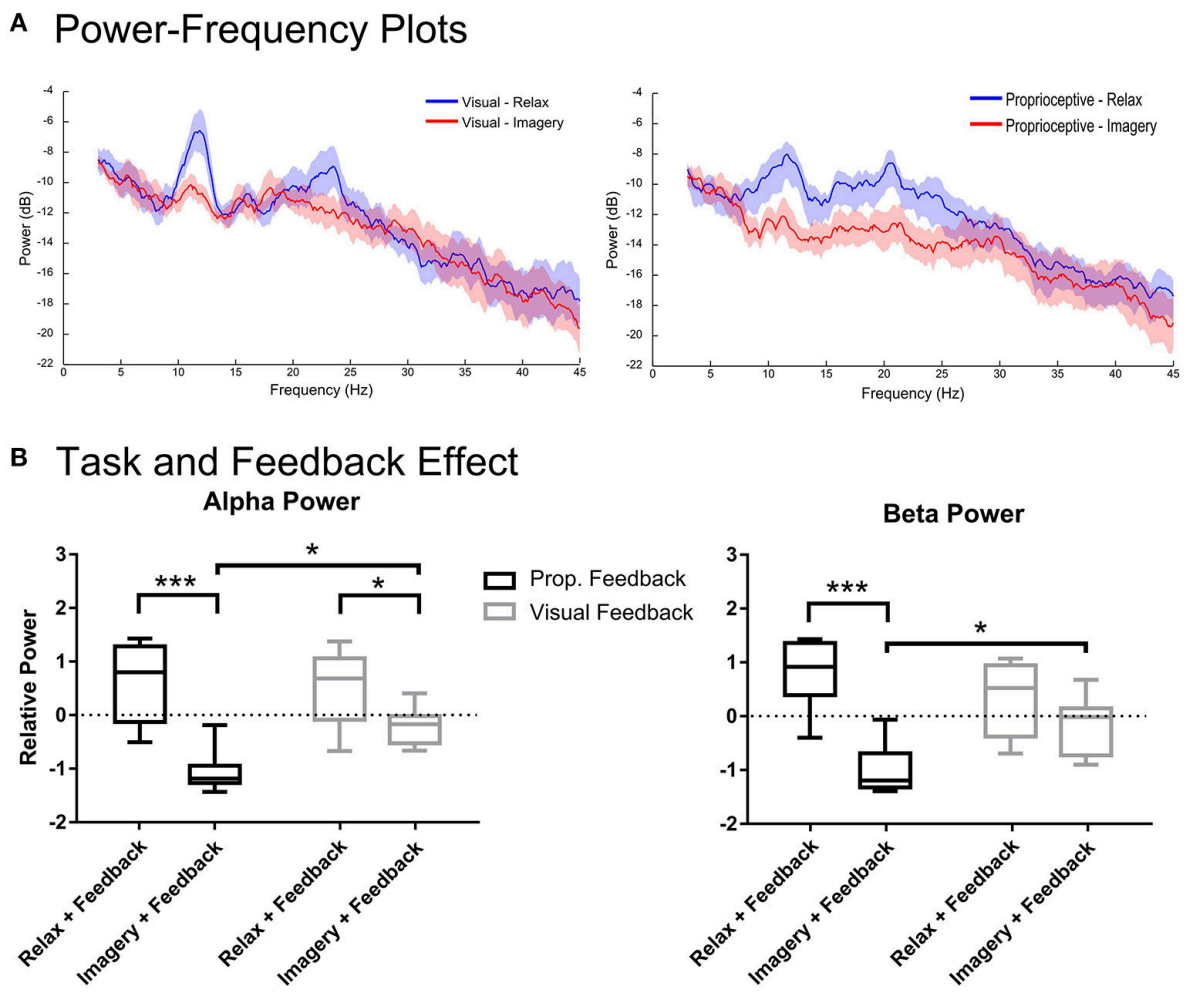
**Spectral analysis of imagery and relaxation with feedback**. Section **(A)** illustrates the log-transformed (10log_10_) spectral power of imagery and relaxation trials during visual or proprioceptive feedback for eight participants. The solid lines represent mean spectral power for each task and their circumscribing shaded area indicates the standard deviation. Section **(B)** presents the results of repeated measures 2-way ANOVA that analyses the task (levels: relaxation and MI) and feedback modality (levels: visual and proprioceptive) effects on the log-transformed and z-cored spectral power in α and β bands for eight participants. The horizontal line in each boxplot, represents the mean value and the lower and upper whiskers depict the minimum and maximum values for each condition, respectively (sample size: 8; ^*^p < 0.05; ^***^p < 0.001; prop: proprioceptive).

**Figure 4:**
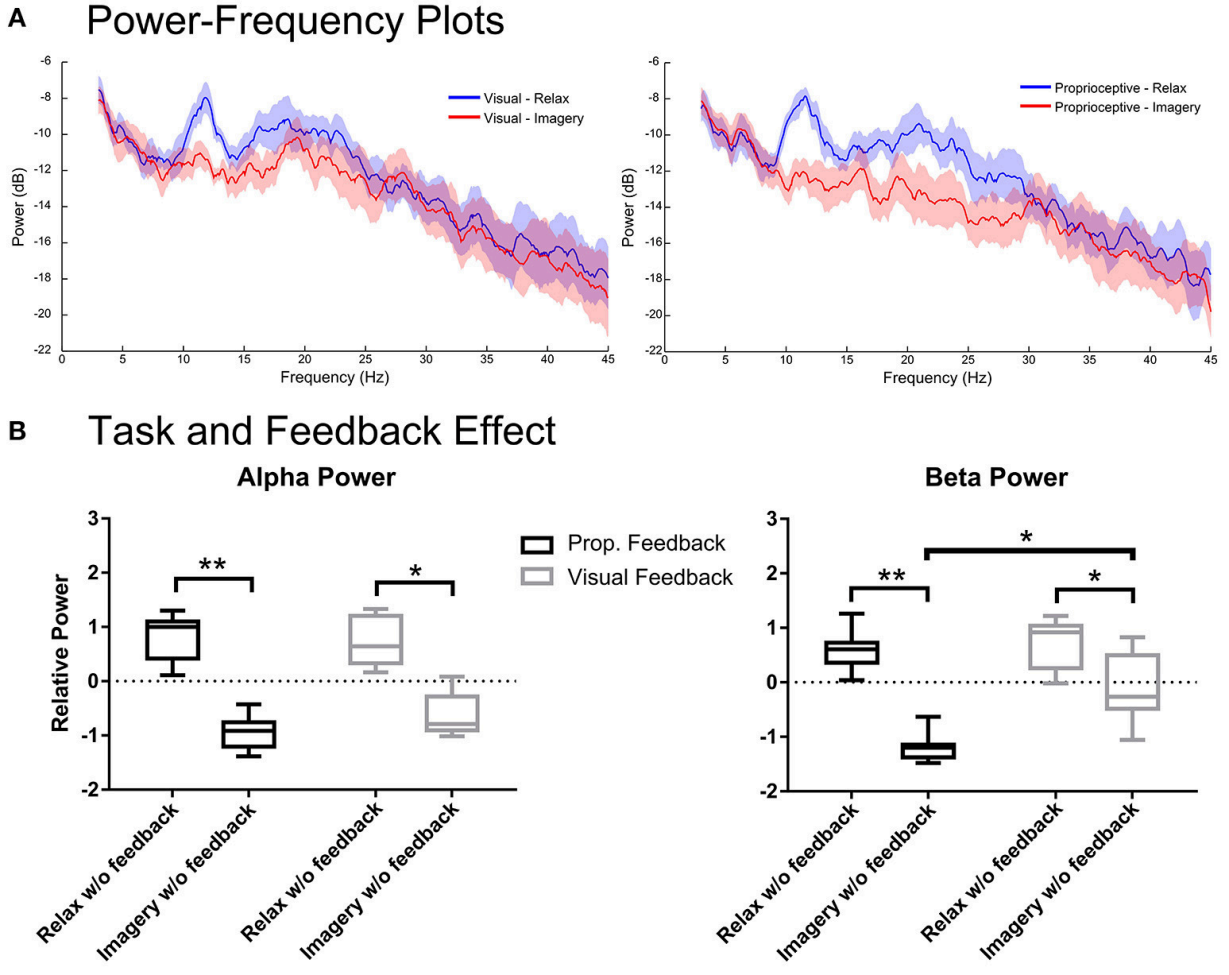
**Spectral analysis of imagery and relaxation ***without*** feedback. (A)** Illustrates the log-transformed (10log_10_) spectral power of imagery/relaxation trials *without* feedback (i.e., during the delay period before visual or proprioceptive feedback onset). The solid lines represent mean spectral power for each task and their circumscribing shaded area indicate the standard deviation. **(B)** Illustrates the results of repeated measures 2-way ANOVA that analyses the task (levels: relaxation and MI) and the modality effects *before* feedback (levels: visual and proprioceptive) onset on the log-transformed and z-cored spectral power in α and β bands for eight participants. The horizontal line in each boxplot, represents the mean value and the lower and upper whiskers depict the minimum and maximum values for each condition, respectively (sample size: 8; ^*^p < 0.05; ^**^p < 0.01; prop: proprioceptive).

**Figure 5:**
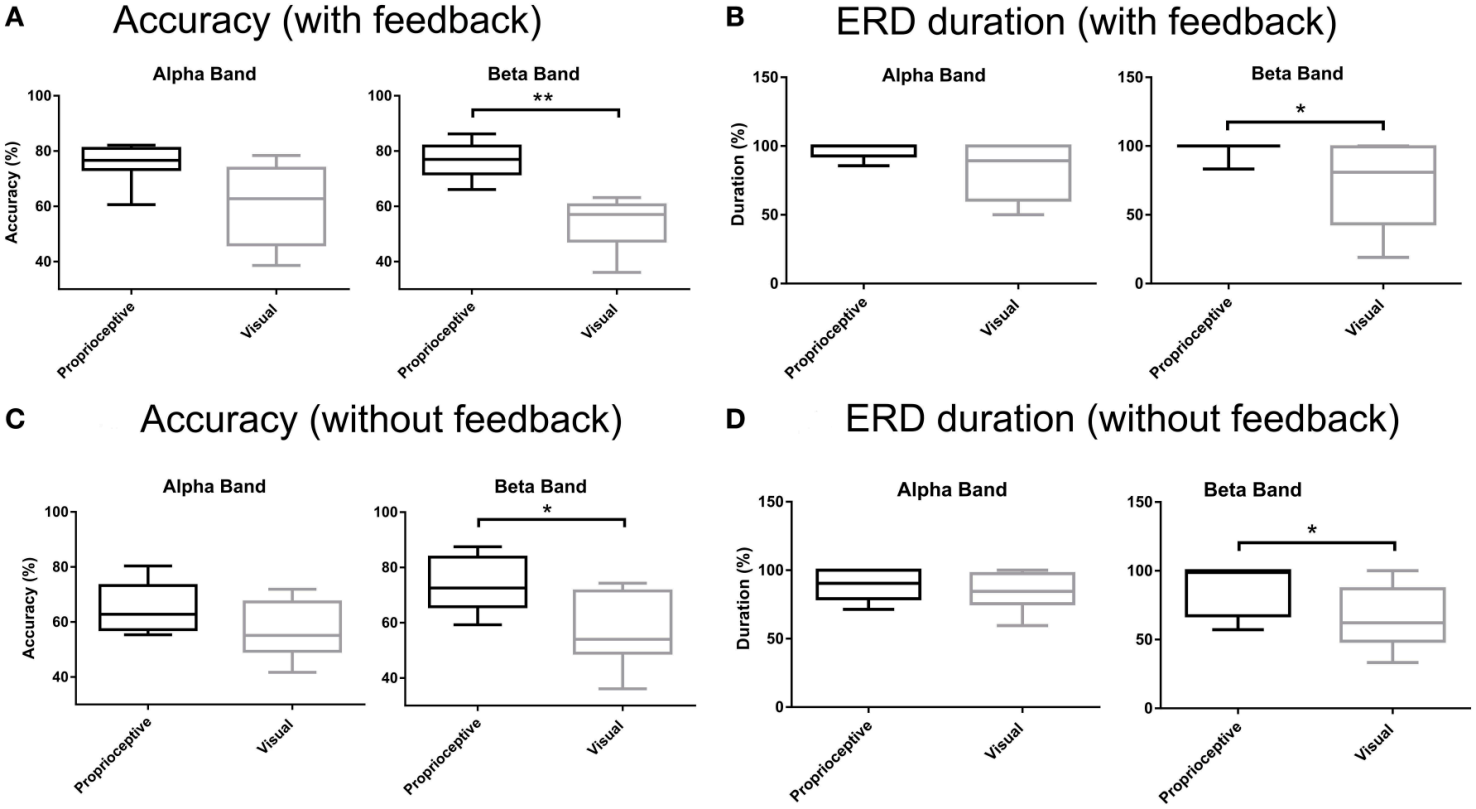
**Accuracy and ERD duration with and without feedback. (A)** Classification accuracy with visual or proprioceptive feedback in α and β bands. **(B)** Event-related desynchronization (ERD) duration with visual or proprioceptive feedback in α and β bands. **(C)** Classification accuracy without visual or proprioceptive feedback in α and β bands. **(D)** ERD duration without visual or proprioceptive feedback in α and β bands. The horizontal line in each boxplot, represents the mean value and the lower and upper whiskers depict the minimum and maximum values for each condition, respectively (^*^p < 0.05; ^**^p < 0.01).

We employed the following indices to compare the effect of visual and proprioceptive feedback on MI performance with and without feedback: (i) the spectral power in α and β-bands during MI/relaxation tasks; (ii) accuracy, i.e., the percentage of trials in which the feedback conformed to the MI task, i.e., equivalent to the classical target hit rate in the cursor position update paradigm, (iii) ERD duration in imagery trials, i.e., the average percentage of times in each trial that the classifier output conformed to the MI task and moved either the orthosis (with proprioceptive condition) or the cursor in the expected direction (with visual condition). The second and third measures were also computed for the subject specific frequencies (Table 2) in addition to the α and β-bands (Figures 3–**5**).

**Table 2:** **Comparison of subject-specific accuracy and ERD duration with and without visual/proprioceptive feedback**.

**Studied conditions**	**Accuracy**			**ERD duration**		
	**Visual (%)**	**Proprioceptive (%)**	***p-*value**	**Visual (%)**	**Proprioceptive (%)**	***p*-value**
With feedback	75	83	0.0011	65	75	<0.0001
Without feedback	60	75	0.0027	63	76	0.0028

To calculate the first measure, offline spectral analysis of the EEG signals was performed with EEGLAB (Delorme and Makeig, 2004) and custom made Matlab scripts. Here, EEG signals were spatially filtered to derive C3-LLP and C4-LLP surrogate channels as in the online processing. For the two participants with the left hand as the target hand, C3-LLP and C4-LLP were swapped resulting in group-wide “contralateral” and “ipsilateral” channels. Channel data were bandpass filtered between 3 and 47 Hz and resampled at 128 Hz. Channel data were segmented into epochs from −2 to 4 s after the auditory command and each trial had its average baseline value subtracted. Outlier trials were rejected using EEGLAB based on signal amplitude, variance, probability, and spectral power according to previous recommendations (Daly et al., 2012). This included signals with amplitudes larger than ±50 μV as they most probably represent either biological (such as muscle artifacts) or environmental noise. In total, 4.1% of trials were rejected. For each retained trial, the spectral band power was integrated over two frequency bands: α (8–13 Hz) and β (16–26 Hz). The spectral power for MI without feedback was calculated for 1 s in the delay period, i.e., in the interval from +1 to +2 s after the auditory command. Accordingly, the spectral power of MI with feedback was estimated for 1 s as well, i.e., from +3 to +4 s after the auditory command.

To re-calculate accuracy a 10-fold cross-validated linear discriminant analysis was used to classify trials as “relaxation” or “imagery.” Predictors included spectral power in contralateral, central and ipsilateral channels. Classification was performed separately for α and β power, and for visual and proprioceptive feedback. Time windows for the “without” and “with” feedback conditions were defined as 1–2 and 3–4 s, respectively.

To re-calculate ERD duration in α and β-bands with and without feedback, the same time windows as for the accuracy (see above) were used. To simulate the real time situation, the spectral power of a 500 ms target window (shifting 24 ms at each step across the 1-s time window) was calculated until the whole time window was swept. This methodology provided a collection of 42 (≈1,000/24) spectral power data for each condition (relaxation/imagery). Then, using the z-score of all spectral data (both relaxation and imagery trials), the spectral data were normalized and only negative values (indicating ERD) were counted. The resultants were divided by 42 and multiplied by 100 to determine offline ERD duration percentage for (MI) trials.

### Statistical analysis

GraphPad Prism version 6.05 was used for statistical analysis. For the accuracy and ERD duration with and without feedback, an unpaired Wilcoxon rank-sum test was applied for statistical analysis. Due to application of multiple comparisons, Bonferroni correction was adopted.

Prior to statistical analysis, the spectral power in α and β-bands was normalized using a z-score transformation, resulting in intra-individual zero-mean and unit-variance spectral data. The normalized spectral data were subjected to a repeated-measures 2-way ANOVA with factors Task (levels “relaxation” and “imagery”) and Feedback Modality (levels “proprioceptive” and “visual”) and two-sided *t*-tests (Holm-Sidak's multiple comparison tests) for *post-hoc* comparisons. The statistical analysis of spectral data was performed separately for with and without feedback conditions.

The data must satisfy a number of assumptions to allow the application of ANOVA. Such assumptions include (i) normality, (ii) homogeneity of variance, and, (iii) sphericity of the dependent factor for different conditions. The first two criteria are soft, which means that ANOVA is robust against some level of their deviation; sphericity, however, is the hard criterion. The applied logarithmic transform that was used to present the spectral power in decibel (dB), made them normally distributed, which was further verified using the Jarque-Bera test. Also, all data blocks showed to be either symmetric or at most mildly asymmetric, i.e., they revealed absolute skewness <1. Regarding homogeneity of variance, the applied z-score transformation that was employed to remove a potential subject-specific bias turned the spectral power into zero-mean and unit-variance data for each participant and thereby minimized the differences in variance among different conditions. The homogeneity of variance for the within-subject factor was verified using calculation of F_max_ (to be <4) for all combinations of data blocks. Finally, since each independent factor (task and feedback modality) had only two levels, sphericity was not a concern and did not need to be checked for a 2-way ANOVA. Therefore, the adopted logarithmic and z-score transformations have turned the data into a valid format for application of a repeated measures 2-way ANOVA.

## Results

### Task and feedback modality effects for with feedback condition

Figure 3 illustrates the spectral power of the 3–4 s post-stimulus period in which either relaxation or (MI) was performed with proprioceptive or visual feedback. There were task-related changes in spectral power in α and β-bands (Figure 3A). The repeated measures 2-way ANOVA analysis of α band revealed a significant main effect for task [*F*_(1, 7)_ = 97.37, *p* < 0.0001] and a significant interaction between task and feedback modality [*F*_(1, 7)_ = 6.128, *p* = 0.0426]. The *post-hoc t*-test (Figure 3B) showed that during both proprioceptive and visual feedback provision the spectral power were significantly larger for relaxation than imagery trials [*t*_(7)_ = 6.09, *p* = 0.0010, and *t*_(7)_ = 2.59, *p* = 0.0358, respectively]. Moreover, feedback modality was indifferent for relaxation trials [*t*_(7)_ = 0.44, *p* = 0.6710], whereas imagery trials produced significantly stronger ERDs with proprioceptive feedback than with visual feedback [*t*_(7)_ = 3.06, *p* = 0.0365]. Similarly, the β-band showed a significant main effect for task [*F*_(1, 7)_ = 62.76, *p* < 0.0001] and a significant interaction between task and feedback modality [*F*_(1, 7)_ = 10.23, *p* = 0.0151]. The *post-hoc t*-test between imagery and relaxation trials (Figure 3B) revealed a significant difference with proprioceptive feedback [*t*_(7)_ = 6.326, *p* = 0.0003] but not for visual feedback [*t*_(7)_ = 1.803, *p* = 0.1145]. Investigation of the impact of feedback modality on the β power (Figure 3B) indicated that imagery with proprioceptive and visual feedback were significantly different [*t*_(7)_ = 2.94, *p* = 0.0429], whereas relaxation trials showed no significant difference between feedback conditions [*t*_(7)_ = 1.582, *p* = 0.1576]. In summary, α ERD was facilitated by both feedback modalities with stronger impact of proprioceptive feedback; whereas β ERD was facilitated by proprioceptive feedback only. In addition, proprioceptive feedback was essential to achieve ERD consistently below baseline (Figure 3B) across both α and β-frequency bands [*t*_(7)_ = 9.017, *p* < 0.0001 for proprioceptive feedback and *t*_(7)_ = 2.225, *p* = 0.1192 for visual feedback].

### Task and feedback modality effects for without feedback condition

Figure 4 shows the spectral power from the 1–2 s period of the proprioceptive and visual conditions during which participants performed the task without feedback. Figure 4A depicts the spectral power in α and β-bands for each feedback modality. Analysis of the α band showed significant main effects only for task [*F*_(1, 7)_ = 840.8, *p* < 0.0001], but neither for feedback [*F*_(1, 7)_ = 1.116, *p* = 0.3258] nor interaction [*F*_(1, 7)_ = 0.7129, *p* = 0.4264]. The *post-hoc t*-test showed that relaxation and imagery trials were significantly different for both proprioceptive [*t*_(7)_ = 4.961, *p* = 0.0033] and visual [*t*_(7)_ = 3.767, *p* = 0.0140] conditions (Figure 4B). However, analysis of the β-band power showed a significant main effect for both task [*F*_(1, 7)_ = 79.68, *p* < 0.0001] and feedback modality [*F*_(1, 7)_ = 11.30, *p* = 0.0121] but revealed no interaction between two factors [*F*_(1, 7)_ = 4.869, *p* = 0.0631]. The *post-hoc t*-test between imagery and relaxation showed a significant difference for both the proprioceptive [*t*_(7)_ = 5.994, *p* = 0.0011] and the visual [*t*_(7)_ = 2.87, *p* = 0.0478] conditions (Figure 4B). Comparing the effect of feedback modality on the β power (Figure 4B) revealed significant differences between conditions for (MI) [*t*_(7)_ = 3.54, *p* = 0.0189], but not for relaxation [*t*_(7)_ = 0.42, *p* = 0.69]. The findings indicate that in both conditions sufficient α and β ERD was achieved, even prior to feedback onset. However, the proprioceptive condition was superior to the visual condition with regard to β-band modulation. Similar to the situation “with feedback,” the proprioceptive condition was essential to keep ERD consistently below baseline (Figure 4B) across both α and β-bands [*t*_(7)_ = 12.00, *p* < 0.0001 for the proprioceptive and *t*_(7)_ = 2.831, *p* = 0.0501 for the visual conditions].

### Accuracy and ERD duration for individual frequencies

Motor imagery (MI) performance with and without feedback were quantified using average accuracy, and the average percentage of ERD duration in each trial. These measures were obtained through calculation of spectral power in each subjects' optimum frequency according to their screening session. Results are summarized in Table 2 where medians of all studied measures and their corresponding *p*-values are reported. For (MI) with feedback, accuracy was 8% higher for the proprioceptive compared to the visual feedback condition (*p* = 0.0011). In addition, ERD duration was also longer with proprioceptive than with visual feedback by 10% (*p* < 0.0001). Considering (MI) without feedback, the proprioceptive input was superior to the visual input for both the accuracy and ERD duration by 15% (*p* = 0.0027), and 13% (*p* = 0.0028), respectively.

### Accuracy and ERD duration for the α and β-bands

Figure 5 depicts the accuracy and duration of ERD with (Figures 5A,B) and without feedback (Figures 5C,D) in α and β-bands. Trial task (“Imagery” vs. “Relaxation”) was classified by linear discriminant analysis using band-power in three channels (ipsilateral, Cz, and contralateral), separately for the α and β-bands, and separately for proprioceptive and visual feedback. According to the paired Wilcoxon rank-sum tests, the accuracy with proprioceptive input was superior to accuracy with visual input for the β-band with (*p* = 0.0078) and without (*p* = 0.0234) feedback. Similarly, ERD duration was longer for the proprioceptive condition as compared to the visual condition for the β-band with (*p* = 0.0313) and without (*p* = 0.0156) feedback. In summary, there was a significantly different behavioral impact for proprioceptive and visual inputs with regard to accuracy and duration of ERD specific to the β-band.

## Discussion

This study demonstrated that MI with proprioceptive feedback was superior to MI with visual feedback to increase the spectral modulation range in α- and β-bands. Moreover, proprioceptive feedback was necessary to consistently achieve MI-related sensorimotor desynchronization (ERD) significantly below baseline. These effects on both the modulation level and MI-related ERD persisted, even in the absence of feedback, thereby indicating fast operant learning of oscillatory self-regulation–which was superior in the proprioceptive as compared to the visual condition. The increased accuracy and duration of learned brain self-regulation achieved in the proprioceptive feedback condition was mediated in the β-band. The particular relevance of β-band oscillations (15–30 Hz) for this approach has been postulated previously (Gharabaghi et al., 2014a,c; Naros and Gharabaghi, 2015) since they mediate the cortico-muscular communication during motor tasks (Riddle and Baker, 2005; Boulay et al., 2011; Witham et al., 2011; Davis et al., 2012; Kilavik et al., 2013) and are linked to the extent of functional impairment after stroke (Rossiter et al., 2014). The findings of the current study also corroborate a recent study (Naros et al., 2016) that demonstrated a link between the operant conditioning of beta desynchronization and subsequent improvements in motor skills via neurofeedback.

Two out of 10 participants were unable to sufficiently modulate their brain activity in this study. This is in line with 15–30% BCI illiteracy reported previously. Recent studies based on mathematical simulation (Bauer and Gharabaghi, 2015a) and empirical evidence (Naros et al., 2016), however, suggest that this limitation might be overcome by novel training regimes which consider the cognitive load (Bauer and Gharabaghi, 2015b), considering the specificity of the feedback (Bauer et al., 2016a) and apply threshold adaptation of the classifier in the course of the intervention (Bauer et al., 2016b).

### Feedback modality and oscillatory modulation range

Previous works showed that neurofeedback training with visual feedback provision increased both laterality (Boe et al., 2014) and movement-associated desynchronization of the targeted β-frequency band for healthy subjects (Bai et al., 2014). Proprioceptive input, however, induced a distributed increase of corticospinal connectivity (Kraus et al., 2016a), involved an extended cortical network including precentral, postcentral and parietal areas (Vukelic et al., 2014) and bridged the gap between individuals' abilities and cortical activations pattern for (MI) and motor execution (Bauer et al., 2015). Moreover, a direct comparison between visual and proprioceptive feedback revealed that the latter was superior the former in activating a distinct cortical network resembling the natural activation during overt movement (Vukelic and Gharabaghi, 2015a). Furthermore, pairing MI-related cortical activity and afferent input increased the corticospinal excitability as further evidence for the critical role of afferent input to improvement in task performance (Mrachacz-Kersting et al., 2012; Niazi et al., 2012; Xu et al., 2014; Gharabaghi et al., 2014a).

Along these lines, the modulation level of the power in α and β-bands in the present study was influenced by the feedback modality as well. The power in the α band, which typically reflects sensorimotor activation and visual information processing (Pfurtscheller, 2001), was modulated by both visual and proprioceptive feedback, with stronger effects of the latter (Figure 3B left). By contrast, the power in the β-band, which is thought to be associated with cortico-muscular communication (Miller et al., 2010; Takemi et al., 2013a,b; Schulz et al., 2014) during MI (Kilavik et al., 2013) and actual movement (McFarland et al., 2000), required proprioceptive input for modulation (Figure 3B right). This significant feedback effect on the modulation range of both α and β-bands was caused by the proprioceptive input on the MI task and not on the relaxation task (Figure 3B). This task-dependency of the feedback effect on cortical activity may be interpreted as follows: (i) The feedback during the relaxation task was visual in both conditions, i.e., observing cursor movement vs. orthosis movement (with no hand involvement). Providing real proprioceptive feedback during the relaxation trials, instead of a modified version of visual feedback (observing orthosis movement), might have resulted in power changes in comparison to the visual feedback condition (observing cursor movement). (ii) The relaxation task might in general be insensitive to the feedback modalities applied in this study. (iii) Proprioceptive input might reveal its effects on cortical physiology during specific brain states only, which are particularly receptive to afferent input, e.g., sensorimotor desynchronization.

Notably, proprioceptive feedback was essential to consistently achieve MI-related sensorimotor ERD significantly below baseline in both α and β-bands. This suggests that coupling proprioceptive feedback with MI provides a better substrate for closing the sensorimotor loop than visual feedback only. This finding is particularly relevant for future rehabilitation applications as ERD has been suggested as a biomarker for corticospinal excitability which is mediated via down-regulation of intracortical inhibition in the human primary motor cortex (Takemi et al., 2013a,b). Furthermore, the amount of β ERD during a MI task with propriceptive feedback has been shown to correlate with robust increases of corticospinal excitability following the intervention (Kraus et al., 2016a). In this context, the present study revealed that rewarding (MI) with contingent proprioceptive feedback will result in stronger (Figure 3B) and longer lasting β ERD (Figure 5B), compared to visual feedback only, and might therefore be especially relevant for restorative approaches (Gharabaghi, 2016; Bauer and Gharabaghi, under review).

### Feedback modality and operant learning

The participants of this study regulated their brain oscillations in both α and β-bands even in those task periods in which they did not receive real-time feedback. This finding might be interpreted in different ways: At first glance, this result might suggest that (i) no feedback at all was necessary to achieve brain self-regulation. However, such a notion would be challenged by several other observations of this study. (ii) Indeed, the feedback phase may also be considered as delayed reward to the phase before feedback onset (i.e., the delay period). (iii) The oscillatory modulation range was relatively larger in the proprioceptive than in the visual condition (Figure 4B). Since the feedback modality in this study was alternating from run to run, i.e., every 15 trials, such a finding would most likely be related to the feedback modality within one run. (iv) Moreover, the ERD remained consistently below baseline only in the proprioceptive condition. (v) There was a significant difference between the β ERD levels of the proprioceptive and visual conditions with stronger desynchronization in the former (Figure 4B). We interpret these converging findings, therefore, as evidence for fast operant learning of oscillatory self-regulation with a stronger impact of the proprioceptive condition. The higher consistency between kinaesthetic MI and proprioceptive feedback (as compared to visual feedback) may have contributed to this finding.

Operant conditioning of neural activity was first demonstrated in animal models (Fetz, 1969, 2007; Ganguly and Carmena, 2009; Engelhard et al., 2013; Hiremath et al., 2015). In humans, the reinforcement learning of self-regulated changes in cortical activity is usually acquired after several training sessions (Zoefel et al., 2011; Florin et al., 2013; Boe et al., 2014; Kaiser et al., 2014). The observed fast operant learning in the present study might be due to different reasons: The delayed feedback onset design may possibly have facilitated brain self-regulation by (i) providing the participants with a preparation period for *ramping* volitional oscillatory modulation (Donoghue et al., 1998; Fetz, 2013), and/or (ii) increasing their reward expectation (Leon and Shadlen, 1999; Savage and Ramos, 2009) during the 2 s lag. Moreover, the interleaved feedback design, switching every 15 trials between proprioceptive and visual feedback, might have caused (iii) sustained attention levels (Lorenz et al., 2014) and/or provided (iv) sufficient novelty to keep up motivation; a moderate level of novelty during learning has been shown to correlate with the highest level of motivation (Heckhausen and Heckhausen, 2008). Finally, applying a specific relaxation task that is (v) rewarded by feedback as well appears to be more able to enhance the modulation of the oscillatory range than a relaxation condition without feedback.

However, even within such an optimized environment for operant learning, the feedback modality seems to play a relevant role, suggesting that proprioceptive input may provide a better mean for self-regulation of sensorimotor rhythms than visual input only.

### β-band and the sensorimotor loop

Optimum frequencies, for which participants received feedback, lay within 12–18 Hz frequency band (Table 1). However, subjects modulated their brain oscillations in both α and β-bands. This might be unexpected at first glance, because successful neurofeedback is known to be frequency-specific (Zoefel et al., 2011; Florin et al., 2013; Naros and Gharabaghi, 2015). However, it has to be considered that this previous frequency-specificity was achieved in the course of several sessions, while the present intervention lasted for one session only. Moreover, the feedback modality was alternated in the present examination, while it remained unchanged in previous studies. Furthermore, recent findings suggest that neurofeedback may not only reinforce the feedback frequency band itself, but may be related to different cortical oscillations as well, thus, suggesting cross-frequency interactions. More specifically, a distributed α network has been shown to regulate the local sensorimotor β activity in a performance dependent way, i.e., with good and poor performers of β-band brain-self-regulation revealing different extents of α network lateralization (Vukelic et al., 2014). Along the same lines, a single neurofeedback session that rewarded spatially selective and spectrally specific cortical activities with proprioceptive feedback, modulated the connectivity of distributed resting state networks of the sensorimotor cortex in different frequency bands (Vukelic and Gharabaghi, 2015b). This is particularly relevant for new rehabilitation strategies, since resting state functional connectivity of the motor cortex seems to be relevant for motor learning (Mottaz et al., 2015) and for prediction of functional improvement after stroke (Nicolo et al., 2015).

In the present study, the feedback modality had a behaviourally relevant impact, as well, by improving the online classification accuracy and duration of brain self-regulation in the proprioceptive as compared to the visual condition (Table 2). Importantly, the proprioceptive input allowed the subjects to achieve an accuracy of > 70% even before feedback onset, a level which is regarded as the threshold for achieving a sense of self-efficacy during operant learning in brain-interface procedures (Kubler et al., 2001). Analysing α and β-bands separately (Figure 5) revealed the relevance of the latter for mediating these performance gains. These findings are in line with the current literature indicating that β-band oscillations mediate the cortico-muscular communication (Riddle and Baker, 2005; Boulay et al., 2011; Witham et al., 2011; Davis et al., 2012; Kilavik et al., 2013), sensorimotor control (Boulay et al., 2011; Brittain et al., 2014) and motor learning (Herrojo Ruiz et al., 2014; Pollok et al., 2014). The particular relevance of β-band oscillations with behavioral gains in the present study is congruent with recent proof of concept data indicating that operant conditioning of β-band ERD will lead to task-specific motor improvement after stroke (Naros and Gharabaghi, 2015). Overall, it suggests the suitability of β-band oscillations as a biomarker for state-dependent stimulation (Gharabaghi et al., 2014a; Kraus et al., 2016b; Raco et al., 2016; Royter and Gharabaghi, 2016) and restorative neuroprosthetics (Gharabaghi et al., 2014b,c; Grimm and Gharabaghi, 2016; Grimm et al., 2016a,b,c) in the context of motor rehabilitation after stroke.

## Conclusion

The present study provided empirical evidence that proprioceptive feedback was superior to visual feedback with regard to the induced strength and duration of ERD as well as the accuracy of task performance. Proprioceptive input provides, therefore, a better environment for operant learning via neurofeedback training. The observed MI-related effects were present not only during real-time feedback provision, but also in the delay period prior to feedback onset. Since the observed effects occurred within only one training session they may be considered as *fast operant learning* of brain self-regulation. Reinforcing β-band ERD via proprioceptive feedback may, therefore, provide a suitable approach for enhancing the sensorimotor loop for rehabilitation that needs to be confirmed in future patient studies.

## Author contributions

SD designed and performed research, analyzed data and wrote the paper. AG analyzed data and wrote the paper. CB analyzed data and wrote the paper. MR performed research and edited the paper. DA performed research and edited the paper. MB designed research, analyzed data and edited the paper.

### Conflict of interest statement

The authors declare that the research was conducted in the absence of any commercial or financial relationships that could be construed as a potential conflict of interest.
